# Infinitely Many Homoclinic Solutions for Second Order Nonlinear Difference Equations with *p*-Laplacian

**DOI:** 10.1155/2014/276372

**Published:** 2014-05-14

**Authors:** Guowei Sun, Ali Mai

**Affiliations:** ^1^Department of Applied Mathematics, Yuncheng University, Yuncheng 044000, China; ^2^School of Mathematics and Information Science, Guangzhou University, Guangzhou, Guangdong 510006, China

## Abstract

We employ Nehari manifold methods and critical point theory to study the existence of nontrivial homoclinic solutions of discrete *p*-Laplacian equations with a coercive weight function and superlinear nonlinearity. Without assuming the classical Ambrosetti-Rabinowitz condition and without any periodicity assumptions, we prove the existence and multiplicity results of the equations.

## 1. Introduction

Difference equations represent the discrete counterpart of ordinary differential equations and are usually studied in connection with numerical analysis. As it is well known, the critical point theory is used to deal with the existence of solutions of difference equations. For example, in 2003, Guo and Yu [1] introduced a variational structure associated with second order difference equations; they employ Rabinowitz's saddle point theorem (see [2]) to obtain the existence of *kT*-periodic solutions for the *T*-periodic system:

(1) $$\begin{array}{c} \Delta^{2}u\left(n-1\right)+\nabla_{u}F\left[n,u\left(n\right)\right]=0,\;n\in Z. \end{array}$$

The forward difference operator Δ is defined by Δ*u*(*n*) = *u*(*n* + 1) − *u*(*n*). They assume that ∇_*u*_ *F* is bounded and *F* is coercive with respect to *u* or *F* satisfies a subquadratic Ambrosetti-Rabinowitz condition and a related coercivity condition. In particular, when *F*(*n*, 0) = 0 for all *n* ∈ *Z*, they prove the existence of nontrivial *kT*-periodic solutions of (1). In [3], they assume that *F* satisfies a superquadratic Ambrosetti-Rabinowitz condition and ∇_*u*_ *F* satisfies a superlinear condition near *u* = 0 and prove the existence of two nontrivial *T*-periodic solutions of (1) by using the similar methods. A survey of those results is given in [4]. In 2004, Zhou et al. [5] consider the case, where the nonlinearity is neither superlinear nor sublinear and generalize the results of [3]. In these papers, the critical point theory is applied to find the periodic solutions of difference equations. The main idea of these papers is to construct a suitable variational structure, so that the critical points of the variational functional correspond to the periodic solutions of the difference equations. Naturally, the critical point theory is also applied to find homoclinic solutions of difference equations; see [6–11] and the reference therein.

In this paper, we consider the following second order nonlinear difference equations with *p*-Laplacian:

(2) $$\begin{array}{c} -\Delta \phi_{p}\left(\Delta u\left(k-1\right)\right)+b\left(k\right)\phi_{p}\left(u\left(k\right)\right)=f\left(k,u\left(k\right)\right),\;k\in Z, \end{array}$$

where *ϕ*_*p*_(*t*) = |*t*|^*p*−2^ *t* for all *t* ∈ *R*, *p* > 1. *b* : *Z* → *R* is a positive and coercive weight function and *f*(*k*, *u*) : *Z* × *R* → *R* is a continuous function on *u*. The forward difference operator Δ is defined by

(3) $$\begin{array}{c} \Delta u\left(k-1\right)=u\left(k\right)-u\left(k-1\right),\;\forall k\in Z. \end{array}$$

As usual, *Z* and *R* denote the set of all integers and real numbers, respectively.

Assume further that *f*(*k*, 0) = 0; then *u*(*k*) ≡ 0 is a solution of (2), which is called the trivial solution. As usual, we say that a solution *u* = {*u*(*k*)} of (2) is homoclinic (to 0): if

(4) $$\begin{array}{c} \underset{\left|k\right|\to \infty }{\lim}u\left(k\right)=0. \end{array}$$

In addition, we are interested in the existence of nontrivial homoclinic solution for (2), that is, solutions that are not equal to 0 identically. In this paper, we also obtain infinitely many homoclinic solutions of (2) for case, where *f* is odd in *u*.

Moreover, we may regard (2) as being a discrete analogue of the following second order differential equation:

(5) $$\begin{array}{c} -{\left(\phi_{p}\left(x^{\prime }\left(t\right)\right)\right)}^{\prime }+b\left(t\right)\phi_{p}\left(x\left(t\right)\right)=f\left(t,x\left(t\right)\right),\;t\in R. \end{array}$$

The study of homoclinic solutions for (2) in case *p* = 2 has been motivated in part by searching standing waves for the nonlinear discrete Schrödinger equation:

(6) $$\begin{array}{c} i{\dot{\psi }}_{n}+\Delta^{2}\psi_{n}-v_{n}\psi_{n}+f\left(n,\psi_{n}\right)=0,\;n\in Z, \end{array}$$

namely, solutions of the form *ψ*_*n*_ = *u*_*n*_ *e* ^−*iωt*^. Periodic assumptions on (6) can be found in [6, 7]. Without any periodic assumptions, the existence and multiplicity of standing wave solutions of (6) are obtained in [8, 9]. We are going to extend the approach of [8] to nonlinear discrete *p*-Laplacian equations.

Throughout this paper, we always suppose that the following conditions hold:

(*B*) function *b* : *Z* → *R* satisfies *b*(*k*) ≥ *b*_0_ > 0 for all *k* ∈ *Z* and
  (7) $$\begin{array}{c} \underset{\left|k\right|\to +\infty }{\lim}b\left(k\right)=+\infty . \end{array}$$
(*f*_1_) *f* ∈ *C*(*Z* × *R*, *R*) and there exist *a* > 0, *q* ∈ (*p*, *∞*), such that
  (8) $$\begin{array}{c} \left|f\left(k,u\right)\right|\leq a\left(1+{\left|u\right|}^{q-1}\right),\;\forall k\in Z,\;u\in R. \end{array}$$
(*f*_2_) lim⁡_|*u*|→0_⁡*f*(*k*, *u*)/|*u*|^*p*−1^ = 0 uniformly for *k* ∈ *Z*.
(*f*_3_) lim⁡_|*u*|→*∞*_⁡*F*(*k*, *u*)/|*u*|^*p*^ = +*∞* uniformly for *k* ∈ *Z*, where *F*(*k*, *u*) is the primitive function of *f*(*k*, *u*); that is,
  (9) $$\begin{array}{c} F\left(k,u\right)=\overset{u}{\underset{0}{\int }}f\left(k,s\right)ds‍. \end{array}$$
(*f*_4_) *u* ↦ *f*(*k*, *u*)/|*u*|^*p*−1^ is strictly increasing on (−*∞*, 0) and (0, *∞*).

In many studies of *p*-Laplacian equations, the following classical Ambrosetti-Rabinowitz superlinear condition ([12, 13]) is assumed:

(10) $$\begin{array}{c} 0<\mu F\left(k,u\right)\leq f\left(k,u\right)u,\;\text{for}\;\text{some}\;\;\mu >p,\;\;u\neq 0. \end{array}$$

It is easy to see that (10) implies *F*(*k*, *u*) ≥ *C*|*u*|^*μ*^, for some constants *C* > 0 and |*u* | ≥1.

In this paper, instead of (10), we assume the *p*-superlinear condition (*f*_3_). It is easy to see that (10) implies (*f*_3_). For example, the *p*-superlinear function,

(11) $$\begin{array}{c} f\left(k,u\right)={\left|u\right|}^{p-2}u\ln\left(1+\left|u\right|\right), \end{array}$$

does not satisfy (10). However, it satisfies the condition (*f*_1_)–(*f*_3_). A crucial role that (10) plays is to ensure the boundedness of Palais-Smale sequences. This is very crucial in applying the critical point theory.

The rest of the paper is organized as follows. In Section 2, we establish the variational framework associated with (2) and then present the main results of this paper.  Section 3 is devoted to prove some useful lemmas, and in Section 4 we prove the main result.

## 2. Preliminaries

We will establish the corresponding variational framework associated with (2).

Consider the real sequence spaces

(12) lp≡lp(Z)={u={u(k)}k∈Z:∀k∈Z,u(k)∈R,||u||lp=(∑k∈Z|u(k)|p)1/p<∞}.

Then the following embedding between *l* ^*p*^ spaces holds:

(13) $$\begin{array}{c} l^{q}\subset l^{p},\;{||u||}_{l^{p}}\leq {||u||}_{l^{q}},\;1\leq q\leq p\leq \infty . \end{array}$$

Define the space

(14) $$\begin{array}{c} E:=\left\{u\in l^{p}:\underset{k\in Z}{\sum }\left[{\left|\Delta u\left(k-1\right)\right|}^{p}+b\left(k\right){\left|u\left(k\right)\right|}^{p}\right]<\infty \right\}. \end{array}$$

Then *E* is a Hilbert space equipped with the norm

(15) $$\begin{array}{c} {||u||}^{p}=\underset{k\in Z}{\sum }\left[{\left|\Delta u\left(k-1\right)\right|}^{p}+b\left(k\right){\left|u\left(k\right)\right|}^{p}\right]. \end{array}$$

|·| is the usual absolute value in *R*.

Now we consider the variational functional *J* defined on *E* by

(16) J(u)=1p∑k∈Z[|Δu(k−1)|p+b(k)|u(k)|p]−∑k∈ZF(k,u(k))=1p||u||p−∑k∈ZF(k,u(k)).

Then *J* ∈ *C* ^1^(*E*, *R*), for all *v* ∈ *E*,

(17) (J′(u),v)=∑k∈Z[ϕp(Δu(k−1))Δv(k−1)+ b(k)ϕp(u(k))v(k)]−∑k∈Zf(k,u(k))v(k),∂J(u)∂u(k)=−Δϕp(Δu(k−1))+b(k)ϕp(u(k))−f(k,u(k)),   k∈Z.

Thus, *u* is a critical point of *J* on *E* only if *u* is homoclinic solutions of (2). We have reduced the problem of finding homoclinic solutions of (2) to that of seeking critical points of the functional *J* on *E*. This means that functional *J* is just the variational framework of (2).

The following lemma plays an important role in this paper; it was established in [11].

**Lemma 1**

If *v* satisfies the condition (*B*), for any *q* > 1, then the embedding map from *E* into *l* ^*q*^(*Z*) is compact.

The main result is as follows.

**Theorem 2**

Suppose conditions (*B*), (*f*_1_)–(*f*_4_) are satisfied. Then we have the following conclusions.

1. Equation (2) has a nontrivial ground state homoclinic solution, that is, homoclinic solutions corresponding to the least positive critical value of the variational functional.
2. If *f*(*k*, *u*) is odd in *u* for each *k* ∈ *Z*, (2) has infinitely many pairs of homoclinic solutions ±*u* ^(*k*)^ in *E*.

To prove the multiplicity results, we need the following lemma.

**Lemma 3 (see [14])**

Let *S* = {*w* ∈ *E* : ||*w*|| = 1}. If *E* is an infinite-dimensional Hilbert space, Φ ∈ *C* ^1^(*S*, *R*) is even and bounded below and satisfies the Palais-Smale condition. Then Φ has infinitely many pairs of critical points.

## 3. Some Useful Lemmas

We define the Nehari manifold:

(18) $$\begin{array}{c} N=\left\{u\in E\setminus \left\{0\right\}:J^{\prime }\left(u\right)u=0\right\}. \end{array}$$

To prove the main results, we need some lemmas.

**Lemma 4**

Suppose conditions (*B*), (*f*_1_)–(*f*_4_) are satisfied. Then, for each *w* ∈ *E*∖{0}, there exists a unique *s*_*w*_ > 0 such that *s*_*w*_ *w* ∈ *N*.

**Proof**

Let *I*(*u*) = ∑_*k*∈*Z*_ *F*(*k*, *u*(*k*)). By (*f*_2_), we have

(19) $$\begin{array}{c} I^{\prime }\left(u\right)=o\left({||u||}^{p-1}\right)\;\text{as}\;\;u⟶0. \end{array}$$

From (*f*_4_), for all *u* ≠ 0 and *s* > 0, we have

(20) $$\begin{array}{c} s⟼\frac{I^{\prime }\left(su\right)u}{s^{p-1}}\;\text{is strictly increasing}. \end{array}$$

Let *W* ⊂ *E*∖{0} be a weakly compact subset and *s* > 0; we claim that

(21) $$\begin{array}{c} \frac{I\left(su\right)}{s^{p}}⟶\infty \;\text{uniformly for}\;\;u\;\;\mathrm{on}\;\;W,\;\text{as}\;\;s⟶\infty . \end{array}$$

Indeed, let {*u*_*n*_} ⊂ *W*. It suffices to show that

(22) $$\begin{array}{c} \text{if}\;\;s_{n}⟶\infty ,\;\frac{I\left(s_{n}u_{n}\right)}{{\left(s_{n}\right)}^{p}}⟶\infty , \end{array}$$

as *n* → *∞*. Passing to a subsequence if necessary, *u*_*n*_⇀*u* ∈ *E*∖{0} and *u*_*n*_(*k*) → *u*(*k*) for every *k*, as *n* → *∞*.

Note that, from (*f*_2_) and (*f*_4_), it is easy to get that

(23) $$\begin{array}{c} F\left(k,u\right)>0,\;\forall u\neq 0. \end{array}$$

Since |*s*_*n*_ *u*_*n*_(*k*)|→*∞* and *u*_*n*_ ≠ 0, by (*f*_3_) and (23), we have

(24) $$\begin{array}{c} \frac{I\left(s_{n}u_{n}\right)}{{\left(s_{n}\right)}^{p}}=\underset{k\in Z}{\sum }\frac{F\left(k,s_{n}u_{n}\left(k\right)\right)}{{\left|s_{n}u_{n}\left(k\right)\right|}^{p}}{\left|u_{n}\left(k\right)\right|}^{p}⟶\infty \;\text{as}\;\;n⟶\infty . \end{array}$$

Therefore, (21) holds.

Let *g*(*s*): = *J*(*sw*), *s* > 0. Then

(25) $$\begin{array}{c} g^{\prime }\left(s\right)=J^{\prime }\left(sw\right)w=s^{p-1}\left({||w||}^{p}-s^{1-p}I^{\prime }\left(sw\right)w\right), \end{array}$$

from (19)–(21); then there exists a unique *s*_*w*_, such that *g*′(*s*) > 0 whenever 0 < *s* < *s*_*w*_, *g*′(*s*) < 0 whenever *s* > *s*_*w*_, and *g*′(*s*_*w*_) = *J*′(*s*_*w*_ *w*)*w* = 0. So *s*_*w*_ *w* ∈ *N*.

**Lemma 5**

Suppose conditions (*B*), (*f*_1_)–(*f*_4_) are satisfied. Then *J* satisfies the Palais-Smale condition on *N*.

**Proof**

Let {*u*_*n*_} ⊂ *N* be a sequence such that *J*(*u*_*n*_) ≤ *d* for some *d* > 0 and *J*′(*u*_*n*_) → 0 as *n* → *∞*.

Firstly, we prove that {*u*_*n*_} is bounded. In fact, if not, we may assume by contradiction that ||*u*_*n*_|| → *∞* as *n* → *∞*. Let *v*_*n*_ = *u*_*n*_/||*u*_*n*_||. Then there exists a subsequence, still denoted by the same notation, such that *v*_*n*_⇀*v*  in  *E* as *n* → *∞*.

Suppose *v* = 0. For every *s* > 0, from Lemma 4, we have

(26) $$\begin{array}{c} d\geq J\left(u_{n}\right)\geq J\left(sv_{n}\right)=\frac{1}{p}s^{p}{||v_{n}||}^{p}-I\left(sv_{n}\right)⟶\frac{1}{p}s^{p}. \end{array}$$

This is a contradiction if $s\geq \sqrt[{p}]{pd}$. Therefore, *v* ≠ 0.

According to (21), we have

(27) $$\begin{array}{c} 0\leq \frac{J\left(u_{n}\right)}{{||u_{n}||}^{p}}=\frac{1}{p}-\frac{I\left(||u_{n}||v_{n}\right)}{{||u_{n}||}^{p}}⟶-\infty ,\;n⟶\infty , \end{array}$$

a contradiction again. Thus, {*u*_*n*_} is bounded.

Finally, we show that there exists a convergent subsequence of {*u*_*n*_}. Actually, there exists a subsequence, still denoted by the same notation, such that *u*_*n*_⇀*u*. By Lemma 1, for any *q* > 1, then

(28) $$\begin{array}{c} u_{n}⟶u\;\text{in }l^{q}\left(Z\right). \end{array}$$

Note that

(29) ||un−u||p=(J′(un)−J′(u),(un−u))+∑k∈Z(f(k,un(k))−f(k,u(k)))×(un(k)−u(k)).

By the weak convergence, the first term on the right hand side of (29) approaches 0 as *k* → *∞*.

By (*f*_1_) and (*f*_2_), it is easy to show that, for any *ɛ* > 0, there exists *c*_*ɛ*_ > 0, such that

(30) $$\begin{array}{c} \left|f\left(k,u\right)\right|\leq ɛ{\left|u\right|}^{p-1}+c_{ɛ}{\left|u\right|}^{q-1},\;\;\left|F\left(k,u\right)\right|\leq ɛ{\left|u\right|}^{p}+c_{ɛ}{\left|u\right|}^{q}. \end{array}$$

By Hölder's inequality, we have

(31) ∑k∈Z(f(k,un(k))−f(k,u(k)))(un(k)−u(k))≤∑k∈Z[ɛ(|un(k)|p−1+|u(k)|p−1)+cɛ(|un(k)|q−1+|u(k)|q−1)]|un(k)−u(k)|≤ɛ(||un||lpp−1+||u||lpp−1)||un−u||lp+cɛ(||un||lpq−1+||u||lpq−1)||un−u||lp.

Combining (28) and the boundedness of {*u* ^(*k*)^}, the above inequality implies

(32) ∑k∈Z(f(k,un(k))−f(k,u(k)))(un(k)−u(k))⟶0as  k⟶∞.

It follows from (29) that *u*_*n*_ → *u* in  *E*. This implies that *J* satisfies the Palais-Smale condition.

## 4. Proof of Main Results

**Proof of Theorem 2**

(1) Now we need five steps to finish this proof.

*Step* *1*. We claim that *N* is homeomorphic to the unit sphere *S* in *E*.

By (19) and (21), *g*(*s*) > 0 for *s* > 0 small and *g*(*s*) < 0 for *s* > 0 large. So *s*_*w*_ is a unique maximum of *g*(*s*) and *s*_*w*_ *w* is the unique point on the ray *s* ↦ *sw* (*s* > 0) which intersects *N*. That is, *u* ∈ *N* is the unique maximum of *J* on the ray. Therefore, by Lemma 4, we may define the mapping $\hat{m}:E\setminus \{0\}\to N$ by setting

(33) $$\begin{array}{c} \hat{m}\left(w\right):=s_{w}w. \end{array}$$

Next we show that the mapping $\hat{m}$ is continuous. Indeed, suppose *w*_*n*_ → *w* ≠ 0. Since $\hat{m}(tu)=\hat{m}(u)$, for each *t* > 0, we may assume *w*_*n*_ ∈ *S* for all *n*. Write $\hat{m}(w_{n})=s_{w_{n}}w_{n}$. Then {*s*_*w*_*n*__} is bounded. If not, *s*_*wn*_ → *∞* as *n* → *∞*.

Note that, by (*f*_4_), for all *u* ≠ 0,

(34) 1pf(k,u)u−F(k,u)=1pf(k,u)u−∫0uf(k,s)ds>1pf(k,u)u−f(k,u)up−1∫0usp−1ds=0.

Therefore, for all *u* ∈ *N*, we have

(35) J(u)=J(u)−1pJ′(u)u=∑k∈Z(1pf(k,u(k))u(k)−F(k,u(k)))>0.

Combining with (*f*_3_) and Lemma 4, we have

(36) 0<J(swnw)(swn)p=1p||w||p−∑k∈ZF(k,swnw(k))|swnw(k)|p|w(k)|p⟶−∞,as  n⟶∞;

this is a contradiction. Therefore, *s*_*w*_*n*__ → *s* > 0 after passing to a subsequence if needed, since *N* is closed and $\hat{m}(w_{n})=s_{w_{n}}w_{n}\to sw,sw\in N$. Hence $sw=s_{w}w=\hat{m}(w)$ by the uniqueness of *s*_*w*_ of Lemma 4. Therefore, $\hat{m}$ is continuous.

Then we define a mapping *m* : *S* → *N* by setting $m:=\hat{m}{|}_{S}$, then *m* is a homeomorphism between *S* and *N*, and the inverse of *m* is given by *m* ^−1^(*u*) = *u*/||*u*||.

*Step* *2*. Now we define the functional $\hat{\Psi }:E\setminus \{0\}\to R$ and Ψ : *S* → *R* by

(37) $$\begin{array}{c} \hat{\Psi }\left(w\right):=J\left(\hat{m}\left(w\right)\right),\;\;\Psi \left(w\right):=\hat{\Psi }{|}_{S}. \end{array}$$

Then we have

$\hat{\Psi }\in C^{1}(E\setminus \{0\},R)$ and Ψ ∈ *C* ^1^(*S*, *R*). Moreover,

(38) $$\begin{array}{c} {\hat{\Psi }}^{\prime }\left(w\right)z=\frac{||\hat{m}\left(w\right)||}{||w||}J^{\prime }\left(\hat{m}\left(w\right)\right)z\;\forall w,z\in E,\;w\neq 0, \end{array}$$

(39) Ψ′(w)z=||m(w)||J′(m(w))z∀z∈Tw(S)={v∈E:(w,v)=0}.

In fact, let *w* ∈ *E*∖{0} and *z* ∈ *E*. By Lemma 4 and the mean value theorem, we obtain

(40) Ψ^(w+tz)−Ψ^(w)=J(sw+tz(w+tz))−J(sww)≤J(sw+tz(w+tz))−J(sw+tz(w))=J′(sw+tz(w+τttz))sw+tztz,

where |*t*| is small enough and *τ*_*t*_ ∈ (0,1). Similarly,

(41) Ψ^(w+tz)−Ψ^(w)=J(sw+tz(w+tz))−J(sww)≥J(sw(w+tz))−J(sw(w))=J′(sw(w+ηttz))swtz,

where *η*_*t*_ ∈ (0,1). From the above, the function *w* ↦ *s*_*w*_ is continuous, combining these two inequalities that

(42) lim⁡t→0Ψ^(w+tz)−Ψ^(w)t=swJ′(sww)z=||m^(w)||||w||J′(m^(w))z.

Hence the Gâteaux derivative of  $\hat{\Psi }$ is bounded linear in *z* and continuous in *w*. It follows that $\hat{\Psi }$ is a class of *C* ^1^ (see [15], Proposition 1.3) and (38) holds. Note only that, since *w* ∈ *S*, $m(w)=\hat{m}(w)$, so (39) is clear.

*Step* *3*. {*w*_*n*_} is a Palais-Smale sequence for Ψ if and only if {*m*(*w*_*n*_)} is a Palais-Smale sequence for *J*.

Let {*w*_*n*_} be a Palais-Smale sequence for Ψ and let *u*_*n*_ = *m*(*w*_*n*_) ∈ *N*. Since for every *w*_*n*_ ∈ *S* we have an orthogonal splitting, *E* = *T*_*w*_*n*__ *S* ⊕ *Rw*_*n*_, we have

(43) ||Ψ′(wn)||=sup⁡z∈TwnS||z||=1Ψ′(wn)z=||m(wn)||sup⁡z∈TwnS||z||=1J′(m(wn))z=||un||sup⁡z∈TwnS||z||=1J′(un)z.

Then

(44) ||Ψ′(wn)||≤||un||||J′(un)||=||un||sup⁡z∈TwnS,t∈Rz+tw≠0J′(un)(z+tw)||z+tw||≤||un||sup⁡z∈TwnS∖{0}J′(un)(z)||z||=||Ψ′(wn)||.

Therefore

(45) $$\begin{array}{c} ||\Psi^{\prime }\left(w_{n}\right)||=||u_{n}||||J^{\prime }\left(u_{n}\right)||. \end{array}$$

By (35), for *u*_*n*_ ∈ *N*, *J*(*u*_*n*_) > 0, so there exists a constant *c* > 0 such that *J*(*u*_*n*_) > *c*. And since *c* ≤ *J*(*u*_*n*_) = (1/*p*)||*u*_*n*_||^*p*^ − *I*(*u*_*n*_)≤(1/*p*)||*u*_*n*_||^*p*^, $||u_{n}||\geq \sqrt[{p}]{pc}$. Together with Lemma 5, $\sqrt[{p}]{pc}\leq ||u_{n}||\leq \sup_{n}||u_{n}||<\infty$. Hence {*w*_*n*_} is a Palais-Smale sequence for Ψ if and only if {*u*_*n*_} is a Palais-Smale sequence for *J*.

*Step* *4*. By (45), Ψ′(*w*) = 0 if and only if *J*′(*m*(*w*)) = 0. So *w* is a critical point of Ψ if and only if *m*(*w*) is a nontrivial critical point of *J*. Moreover, the corresponding values of Ψ and *J* coincide and inf⁡_*S*_Ψ = inf⁡_*N*_ *J*.

*Step* *5*. Ψ satisfies the Palais-Smale condition.

Let {*w*_*n*_} be a Palais-Smale sequence for Ψ; then {*u*_*n*_} is a Palais-Smale sequence for *J* by Step 3, where *u*_*n*_ : = *m*(*w*_*n*_) ∈ *N*. From Lemma 5, *u*_*n*_ → *u* after passing to a subsequence and *w*_*n*_ → *m* ^−1^(*u*), so Ψ satisfies the Palais-Smale condition.

Let {*w*_*n*_} ⊂ *S* be a minimizing sequence for Ψ. By Ekeland's variational principle we may assume Ψ′(*w*_*n*_) → 0 as *n* → *∞*, so {*w*_*n*_} is a Palais-Smale sequence for Ψ. By the Palais-Smale condition, *w*_*n*_ → *w* after passing to a subsequence if needed. Hence *w* is a minimizer for Ψ and therefore a critical point of  Ψ; then *u* = *m*(*w*) is a critical point of *J* and also is a minimizer for *J*. Therefore, *u* is a ground state solution of (2).

(2) If *f*(*k*, *u*) is odd in *u* for each *k* ∈ *Z*, then *J* is even, so is Ψ. Since inf⁡_*S*_Ψ = inf⁡_*N*_ *J* > 0 and Ψ satisfies the Palais-Smale condition, Ψ has infinitely many pairs of critical points by Lemma 3. It follows that (2) has infinitely many pairs of homoclinic solutions ±*u*_*n*_ in *E*.

This completes Theorem 2.

Finally, we exhibit examples to demonstrate the applicability of Theorem 2.

**Example 6**

Consider the second order difference equation:

(46) $$\begin{array}{c} -\Delta^{2}u\left(k-1\right)+b\left(k\right)u\left(k\right)=f\left(k,u\left(k\right)\right),\;k\in Z, \end{array}$$

where *b* : *Z* → (0, *R*) such that lim⁡_|*k*|→+*∞*_⁡*b*(*k*) = +*∞*. Let

(47) $$\begin{array}{c} f\left(k,u\right)=u\ln\left(1+\left|u\right|\right), \end{array}$$

for all *k* ∈ *Z*. Then it is clear that all conditions of Theorem 2 are satisfied. By Theorem 2, (46) has infinitely many pairs of homoclinic solutions.

**Example 7**

Consider the *p*-Laplacian difference equation:

(48) −Δ(|Δu(k−1)|p−2Δu(k−1))  +b(k)|u(k)|p−2u(k)=f(k,u(k)), k∈Z,

where *b* : *Z* → (0, *R*) such that lim⁡_|*k*|→+*∞*_⁡*b*(*k*) = +*∞*. Let

(49) f(k,u)={0,u=0,|u|p−2u1−ln⁡|u|,0<|u|≤1,|u|p−2u(1+ln⁡|u|),|u|>1,

for all *k* ∈ *Z* and *p* > 1. It is easy to verify that *f*(*k*, *u*) satisfies all conditions in Theorem 2. Therefore, (48) has infinitely many pairs of homoclinic solutions.
